# Transdiagnostic evaluation of epigenetic age acceleration and burden of psychiatric disorders

**DOI:** 10.1038/s41386-023-01579-3

**Published:** 2023-04-17

**Authors:** Natan Yusupov, Linda Dieckmann, Mira Erhart, Susann Sauer, Monika Rex-Haffner, Johannes Kopf-Beck, Tanja M. Brückl, Darina Czamara, Elisabeth B. Binder

**Affiliations:** 1 Department of Translational Research in Psychiatry, Max Planck Institute of Psychiatry, Munich, 80804 Germany; 2 International Max Planck Research School for Translational Psychiatry (IMPRS-TP), Munich, Germany; 3 Department of Psychology, Ludwig Maximilian University of Munich, Munich, Germany

**Keywords:** Diagnostic markers, Psychiatric disorders

## Abstract

Different psychiatric disorders as well as exposure to adverse life events have individually been associated with multiple age-related diseases and mortality. Age acceleration in different epigenetic clocks can serve as biomarker for such risk and could help to disentangle the interplay of psychiatric comorbidity and early adversity on age-related diseases and mortality. We evaluated five epigenetic clocks (Horvath, Hannum, PhenoAge, GrimAge and DunedinPoAm) in a transdiagnostic psychiatric sample using epigenome-wide DNA methylation data from peripheral blood of 429 subjects from two studies at the Max Planck Institute of Psychiatry. Burden of psychiatric disease, represented by a weighted score, was significantly associated with biological age acceleration as measured by GrimAge and DunedinPoAm (R2-adj. 0.22 and 0.33 for GrimAge and DunedinPoAm, respectively), but not the other investigated clocks. The relation of burden of psychiatric disease appeared independent of differences in socioeconomic status and medication. Our findings indicate that increased burden of psychiatric disease may associate with accelerated biological aging. This highlights the importance of medical management of patients with multiple psychiatric comorbidities and the potential usefulness of specific epigenetic clocks for early detection of risk and targeted intervention to reduce mortality in psychiatric patients.

## Introduction

Psychiatric disorders as well as their risk factors, such as exposures to adverse life events, including childhood adversity or chronic psychosocial stress, have been associated with increased prevalence of age-related diseases and mortality in large meta-analyses [1–4]. Patients with psychiatric disorders have a significantly reduced life expectancy [5]. Understanding of the mechanisms contributing to this risk and availability of biomarkers would contribute to early identification of patients at risk, improved medical management and reduction of overall mortality. Epigenetic information, namely DNA methylation (DNAm), was used to develop so-called ‘epigenetic clocks’ that may serve as indicators of risk for age-related disease and mortality and allow earlier prevention and intervention [6, 7].

Epigenetic clocks were initially developed to accurately estimate chronological age from predictable changes over aging in DNAm patterns, also referred to as ‘DNAm age’ or ‘epigenetic age’ [8]. Deviations of estimated ‘epigenetic age’ from chronological age are termed epigenetic ‘age acceleration’ or ‘age deceleration’ if the estimated epigenetic age is older or younger than the chronological age, respectively. This deviation was shown to better predict a broad range of health outcomes and mortality risks than chronological age or epigenetic age estimation alone [9]. Different epigenetic clocks rely on different DNAm profiles and likely represent different, not yet fully understood, biological mechanisms underlying the aging process [7, 10, 11].

Epigenetic clocks are considered to capture both chronological and biological information, and have been used to investigate health and disease [6]. DNAm phenotypic age estimator (PhenoAge) and DNAm GrimAge [12, 13], tools developed more recently, are referred to as ‘biological clocks’, as opposed to the first ‘chronological clocks’ [8, 14, 15]. These clocks are supposed to represent inter-individual variability contributing to functional decline and disease with age [16], because they were trained on various age-related biological and health-related measures rather than age alone [7, 12, 13]. PhenoAge was constructed from a weighted average of chronological age and nine additional clinical biomarkers (full list in supplementary methods, [12]). GrimAge was constructed from chronological age, sex, DNAm-based surrogates for smoking pack years and seven plasma proteins (full list in supplementary methods, [13]). The Dunedin Pace of Aging Methylation (DunedinPoAm), a modification of the former ‘Pace of aging’ measurement [17, 18], was developed using longitudinal data, which enabled investigation of change in 18 age-associated biomarkers over time within individuals and represents the ‘pace’ rather than the ‘state’ of biological aging (BA).

Many factors influence aging trajectories and several studies have reported accelerated epigenetic age with psychiatric disorders and in individuals exposed to adversity [19]. Epigenetic age acceleration (AgeAccel) has been shown mainly in individuals suffering from posttraumatic stress disorder (PTSD) [20–27] and depression [28], but also in bipolar disorder [29] or anxiety-related disorders [30] and in individuals exposed to risk factors for psychiatric illness, such as childhood adversity [24, 31–33] or cumulative lifetime stress [34–36].

So far, most studies have focused on single psychiatric disorders and not always assessed exposures to adversity. However, psychiatric comorbidity is the rule rather than the exception and exposure to adversity is frequent [37]. It remains unclear whether there are cumulative effects of comorbidity and co-exposure to adversity on BA. In addition, some have proposed analysis using multiple clocks in the same cohorts to explore different components of BA, not captured with single clocks [7, 11].

Our aim was to evaluate the relation of ‘burden of psychiatric disease’, i.e., a weighted score of psychiatric diagnoses derived from a structured diagnostic interview to five different epigenetic clocks (Horvath, Hannum, PhenoAge, GrimAge and DunedinPoAm) calculated from DNAm measured in peripheral blood cells from transdiagnostic samples, including healthy controls. We additionally evaluated how this relationship is altered with exposure to adversity in childhood, and cumulative stress later in life.

## Methods and materials

### Study population

The study sample (*N* = 429) included subjects with psychiatric disorders and self-reported healthy controls who consented for the participation of two studies conducted at the Max Planck Institute of Psychiatry (MPIP) in Munich, Germany: the Biological Classification of Mental Disorders study (BeCOME, registered on ClinicalTrials.gov, TRN: NCT03984084, *N* = 308) [38] and a subset of patients recruited for major depression from a clinical psychotherapy study (OPTIMA, registered on ClinicalTrials.gov, TRN: NCT03287362; *N* = 121) [39] who agreed to participate in an additional biobanking project. Demographic information including age, sex, ethnicity and socioeconomic status was collected using self-reports as stated in the study protocols (see 38, 39 for inclusion and exclusion criteria). Socioeconomic data available for both studies included school education and household income per month (see Table 1). Self-reported data on somatic diseases, including metabolic, cardiovascular, respiratory, immunological and other diseases, was available for 342 participants of both studies (BeCOME=270, OPTIMA=72) and was evaluated as a cumulative score (full list of items in supplementary methods).

**Table 1:** Demographics of cohorts.

	BeCOME (*N* = 301)	OPTIMA (*N* = 119)	Overall (*N* = 420)
Age (years)			
Mean (SD)	35.3 (12.1)	42.9 (13.5)	37.4 (12.9)
Median [Min, Max]	31.8 [18.7, 66.2]	44.7 [19.2, 73.6]	34.0 [18.7, 73.6]
Sex			
Female	193 (64.1%)	65 (54.6%)	258 (61.4%)
Male	108 (35.9%)	54 (45.4%)	162 (38.6%)
Ethnicity			
African	1 (0.3%)	0 (0%)	1 (0.2%)
Asian	6 (2.0%)	0 (0%)	6 (1.4%)
Caucasian	267 (88.7%)	94 (79%)	361 (86.0%)
Hispanic/Latin-american	3 (1.0%)	0 (0%)	3 (0.7%)
Oriental	7 (2.3%)	0 (0%)	7 (1.7%)
Other	10 (3.3%)	0 (0%)	10 (2.4%)
Unknown	2 (0.7%)	0 (0%)	2 (0.5%)
Missing	5 (1.7%)	25 (21.0%)	30 (7.1%)
BMI (kg/m^2^)			
Mean (SD)	23.8 (4.57)	26.6 (5.97)	24.6 (5.16)
Median [Min, Max]	22.7 [15.4, 47.6]	25.6 [17.3, 52.9]	23.2 [15.4, 52.9]
Missing	8 (2.7%)	0 (0%)	8 (1.9%)
Smoking status			
Current smoker	1 (0.3%)	4 (3.4%)	5 (1.2%)
Former smoker	165 (54.8%)	90 (75.6%)	255 (60.7%)
Never smoker	135 (44.9%)	25 (21.0%)	160 (38.1%)
Childhood maltreatment			
Mean (SD)	39.7 (14.4)	43.8 (15.3)	40.9 (14.8)
Median [Min, Max]	35.0 [25.0, 88.0]	41.0 [25.0, 96.0]	37.0 [25.0, 96.0]
Missing	58 (19.3%)	22 (18.5%)	80 (19.0%)
Lifetime stress			
Mean (SD)	19.9 (13.0)	-	19.9 (13.0)
Median [Min, Max]	18.0 [0, 59.0]	-	18.0 [0, 59.0]
Missing	58 (19.3%)	119 (100%)	177 (42.1%)
Burden of psychiatric disease			
Mean (SD)	5.87 (5.53)	9.37 (5.02)	6.89 (5.61)
Median [Min, Max]	4.0 [0, 33.0]	9.0 [2.0, 24.0]	6.0 [0, 33.0]
Missing	21 (7.0%)	4 (3.4%)	25 (6.0%)
School education			
12-13 years	200 (66.4%)	45 (37.8%)	245 (58.3%)
12 years	20 (6.6%)	17 (14.3%)	37 (8.8%)
10 years	32 (10.6%)	17 (14.3%)	49 (11.7%)
9 years	14 (4.7%)	19 (16.0%)	33 (7.9%)
No school graduation	0 (0%)	3 (2.5%)	3 (0.7%)
Still in school	1 (0.3%)	0 (0%)	1 (0.2%)
Other	5 (1.7%)	5 (4.2%)	10 (2.4%)
Missing	29 (9.6%)	13 (10.9%)	42 (10.0%)

Childhood maltreatment was evaluated with CTQ. Lifetime stress was evaluated with MEL and was available only in the BeCOME cohort. Burden of psychiatric disease was evaluated with the computer-based modified version of the Munich-Composite International Diagnostic Interview (DIA-X/M-CIDI).

All participants provided written informed consent. The studies, all procedures, specific sample and data withdrawal request for our research from the MPIP Biobank were approved by the LMU ethics review board.

### Measures

#### Childhood maltreatment

Childhood maltreatment (CM) was assessed with the short version of the Childhood Trauma Questionnaire (CTQ), a retrospective self-report of exposure to different types of abuse and neglect experiences (details in supplementary methods, [40, 41]). Reported CM was summed to a total score of cumulative CM (Table 1, [42]). Additionally, for each subject, the subscales of abuse (emotional, sexual, physical) or neglect (physical, emotional) were categorized into four levels - none, mild, moderate or severe as previously described [41], and operationalized dichotomously (exposed vs. non-exposed). Participants were defined as exposed if moderate or severe exposure was reported in any subscale (details in supplementary methods). To increase power for the interaction analysis, participants who answered ‘no’ in the M-CIDI trauma section for two types of childhood abuse were included in the non-exposed group (*N* = 51 for physical and *N* = 48 for sexual abuse). Final data was obtained for *N* = 391 for physical abuse and *N* = 388 for sexual abuse.

#### Lifetime stress

Lifetime exposure to critical life events (other than CM) was assessed with the short version of the self-reported Munich Event-Questionnaire (MEL) consisting of 27 items covering potentially stressful events from different areas of life and their frequencies (questionnaire only available for the BeCOME cohort) [43]. A total score was calculated using the number of events weighted by their frequencies (Table 1, details in supplementary methods).

#### Burden of psychiatric disease

Participants underwent a modified version of the computer-based Munich-Composite International Diagnostic Interview (M-CIDI) (DIA-X/M-CIDI, [44]) conducted by trained study assistants (details in [38, 39]) to asses current (last four weeks) or past lifetime DSM-IV diagnosis [45]. Individuals could fulfill criteria for no, subthreshold or full diagnoses. Subthreshold diagnoses were defined as fulfilling all but one criterium required for a full diagnosis. Diagnoses were transferred to ICD-10 system definitions [44, 46] and diagnoses of eating and somatoform disorders were excluded, since these were not obtained for all participants. To generate the burden of psychiatric disease score, two points were given for each full diagnosis and one point for each subthreshold diagnosis (Fig. S1). To avoid overrepresentation of specific phobias, the presence of any rather than each specific phobia was counted as one diagnosis (Fig. S2 for percentage of diagnostic categories).

### DNA methylation

Peripheral whole blood samples were drawn from BeCOME and OPTIMA subjects who consented for the MPIP Biobank (ethic committee application number 338-15). DNA was extracted according to standard procedures. Samples were randomized with regard to sex, age, child maltreatment and self-reported case-control status using the *Omixer* R package [47] in a 96-well format before DNA extraction. Bisulfite-conversion of 400 ng DNA was performed with the EZ-96 DNA Methylation kit (Zymo Research, Irvine, CA). Illumina Infinium MethylationEPIC BeadChip (Illumina, San Diego, CA, USA) was used for epigenome-wide methylation analysis of 429 samples according to manufacturer protocols. Methylation data was preprocessed using a standard pipeline [48] with the *minfi* R package [49]. After loading raw intensity values directly into R version 4.0.4 [50] and transformation into beta-values, quality control was performed (*N* = 865,859 probes remained). Samples with a mean detection *p* value > 0.05 (*n* = 7), samples presenting with distribution artefacts in raw beta-values (*n* = 0) or sex mismatches (*n* = 1) were excluded. Normalization was performed using stratified quantile normalization [51] and subsequently beta-mixture quantile normalization (BMIQ, [52]). After transforming beta-values into M-values, we performed a principal component analysis to exclude one outlier (>3 SD on two first principal components). Next, we corrected batch effects sequentially with ComBat within the *sva* R package [53] for the strongest associations with the principal components (plate, array and row). Batch-corrected M-values were transformed into beta-values and MixupMapper [54] confirmed that no sample mix-ups or swaps occurred. The final sample included DNAm data from 420 individuals. Data have been deposited in the Gene Expression Omnibus database (GEO) with the primary accession: GSE222468.

### Genotyping and population stratification

Genotyping was conducted using Illumina global screening arrays (GSA-24v3-0, Illumina, San Diego, CA, US). SNPs with a call rate below 98%, a minor allele frequency below 1% or deviation from Hardy-Weinberg-Equilibrium (*p*-value < 1 × 10^−05^) were excluded. Furthermore, individuals presenting with call rates below 98% were excluded. Only unrelated individuals were included. After LD-pruning, the main multi-dimensional scaling (MDS) components from the IBS matrix were retrieved. Samples with outliers on MDS components (>4 SD on any of the first 10 axes) and heterozygosity outliers (>4 SD) were removed. After QC, genotype data was available for 421 subjects.

### Calculation of epigenetic age and epigenetic age acceleration

DNAmAge of following DNAm clocks: Horvath [8], Hannum [15], PhenoAge [12], GrimAge [13] and their corresponding AgeAccel: AgeAccelHorvath, AgeAccelHannum, AgeAccelPheno and AgeAccelGrim were calculated on normalized batch-corrected beta values with Horvaths’ New Methylation Age Calculator (https://dnamage.genetics.ucla.edu/new, 8). Using the advanced analysis option, cell type proportions (CD8T, CD4T, NK, Bcell, Mono, Gran) were calculated as suggested by Houseman et al. [55, 56]. DunedinPoAm was calculated with the *DunedinPoAm38* R package (https://github.com/danbelsky/DunedinPoAm38, 18). AgeAccel (residuals from a regression of estimated epigenetic age on chronological age) was used for further analysis. ‘Age acceleration’ (positive score) or ‘age deceleration’ (negative score) of an individual were defined according to the direction of the deviation (Fig. S3).

### Statistical analysis

**Fig. 1:**
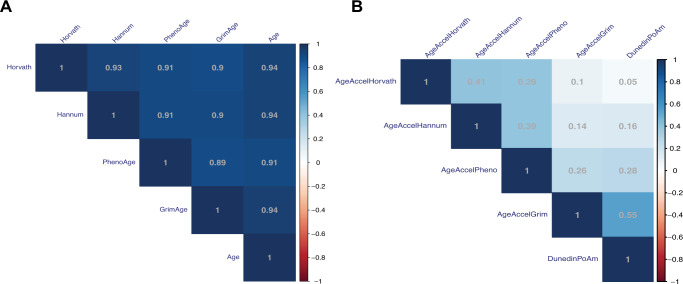
Correlograms of DNA methylation age estimations and accelerations of four DNA methylation clocks and DunedinPoAm. **A** Pearson correlation coefficients (r) of estimated DNA methylation age by different DNA methylation clocks and age. **B** Pearson correlation coefficients (r) of calculated DNA methylation age acceleration by four different DNA methylation clocks and DunedinPoAm.

Pearson correlations were calculated between chronological age and each of the calculated DNAmAge estimations as well as among AgeAccel and DunedinPoAm. Median absolute difference was calculated as suggested by Horvath [8] for each of the DNA methylation age estimators and was within the accepted range (see supplementary methods). The effect of burden of psychiatric disease as independent variable on five measurements of biological age: AgeAccelHorvath, AgeAccelHannum, AgeAccelPheno, AgeAccelGrim and DunedinPoAm as dependent variables was investigated with multiple regression modeling. All models were adjusted for study cohort and covariates that had previously been shown to be associated with AgeAccel: sex [8, 12, 15, 28, 57], ethnicity [57, 58], smoking status [12, 59, 60], body mass index (BMI) [9, 12, 28, 60] and cell type proportions [61]. Since AgeAccel is by definition already adjusted for age, chronological age was added as a covariate only to the DunedinPoAm model. If a covariate had already been used in the training of the epigenetic clock, it was excluded from the specific model (sex and smoking in GrimAge and BMI in DunedinPoAm). For the interaction analysis, an interaction term of burden of psychiatric disease with physical or sexual abuse was included to predict DunedinPoAm. Cell type proportions were intercorrelated (Fig. S4), so including all in one model led to high variance inflation. Granulocytes were dropped from the model, since they were correlated with most other cell types and displayed the highest value (GVIF = 158.53). Smoking status was predicted using the *EpiSmokEr* R package [62]. P-values of different intercorrelated AgeAccel regression models (Fig. 1B) were adjusted for multiple testing based on estimated effective number of tests using the Galwey method within the *poolr* R package [63, 64] resulting in four independent tests of the main analyses. We used stringent Bonferroni correction to correct for the two types of abuse (sexual and physical) and the two DNAm clocks. This is rather stringent given that sexual and physical abuse as well as the two DNAm clocks are correlated (r_s_ = 0.39 and r = 0.55). Beta estimates, standard error and adjusted R^2^ are reported. The first two MDS components of genotype array data were included to account for population stratification. Burden of psychiatric disease was right-skewed and therefore square root transformed when used as an outcome. All statistical analyses were conducted in R version 4.0.4 [50].

## Results

### Prediction of chronological age and DNA methylation age acceleration

DNAmAge estimation of all investigated clocks strongly correlated with chronological age (r = 0.91–0.94) and among the different estimates themselves (r = 0.89–0.93, Fig. 1A, Fig. S5). Correlations of DNAmAge acceleration (AgeAccel), however, varied between the different estimators, showing only a moderate correlation among the two ‘chronological clocks’ and AgeAccelPheno and even less correlation of these with AgeAccelGrim and DunedinPoAm (Fig. 1B).

DunedinPoAm showed a weak positive correlation with AgeAccelHannum (r = 0.16, *p* = 0.001) and AgeAccelPheno (r = 0.28, *p* = 5.977 × 10^−09^) and the overall strongest positive correlation with AgeAccelGrim (r = 0.55, *p* < 2.988 × 10^−34^) (Figure S6). Hence, individuals with advanced ‘biological age’ (‘state of aging’, calculated with AgeAccelGrim) also presented a faster ‘pace of aging’.

Even though all clocks strongly correlated with chronological age, the number of overlapping CpGs for DNA age estimation is very low, at most 35 CpGs between PhenoAge and the Horvath clock (Fig. S7).

### Association of burden of psychiatric disease with DNAm age acceleration and DunedinPoAm

**Fig. 2:**
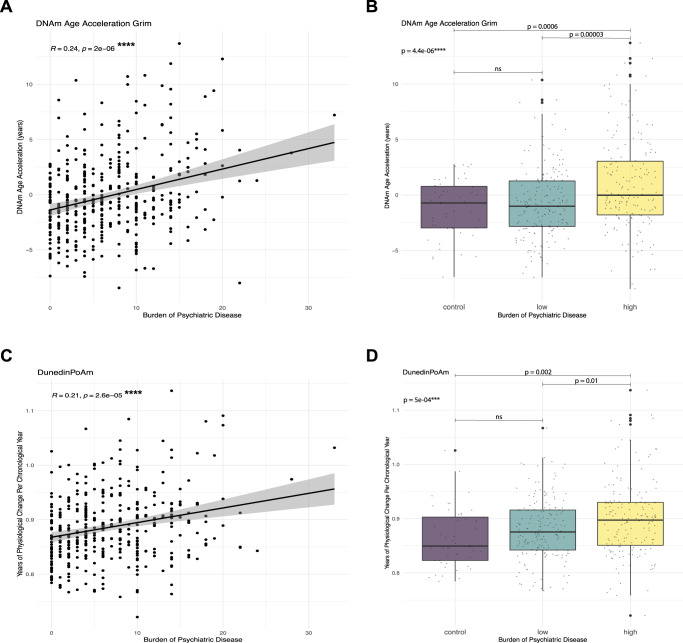
Associations between burden of psychiatric disease and AgeAccelGrim or DunedinPoAm. Spearman correlation between AgeAccelGrim (years) (**A**) or DunedinPoAm (**C**) on the y-axis and burden of psychiatric disease on x-axis (Spearman correlation coefficient, r_s_). **B**, **D** display box plots and *p*-values from an analysis of variance and the corresponding adj. *p*-values from a post hoc Tukey test after categorizing subjects by status of burden of psychiatric disease – control (no current subthreshold or full diagnosis, *N* = 47), low (*N* = 182) and high (*N* = 166) categorized by the median score of participants with score > 0 (median = 7). Data was missing for 25 participants. ****p ≤ 0.0001, ***p ≤ .001, **p ≤ .01; *p ≤ .05. ns not significant.

AgeAccelGrim and DunedinPoAm, but not the other clocks, showed significant positive bivariate associations with burden of psychiatric disease (r^s^ = 0.24, *p* = 2 × 10^−06^ and r_s_ = 0.21, *p* = 2.6 × 10^−05^ respectively, Fig. 2A, C). This correlation remained significant when accounting for the covariates described in the methods section in multiple linear regressions for AgeAccel measured by the different DNAm clocks. Burden of psychiatric disease was significantly associated with AgeAccelGrim (β = 0.155, SE = 0.032, t = 4.81, *p* = 2.24 × 10^−06^, p-adj = 8.96 × 10^−06^, R^2^-adj. = 0.216) and DunedinPoAm (β = 0.002, SE = 0.0005, t = 3.19, *p* = 0.0015, p-adj. = 0.006, R^2^-adj. 0.33, Table 2). We thus focused on these two clocks for all further investigations. Post hoc stratification by sex showed the same direction of effects in both sexes (see Table S1 and Table S2). Excluding participants with extreme burden of psychiatric disease scores (>4 SD = 22.4 of the median=6, *N* = 3) did not change the results with regards to effect, direction and significance. Usage of psychiatric medication had no significant association (*p* = 0.72 & *p* = 0.61, yes = 106 vs. no = 297) and did not change the results with regards to effect, direction and significance. Somatic disease score showed a significant association with DunedinPoAm (β = 0.004, SE = 0.0016, t = 2.598, R2-adj.=0.33, *p* = 0.009, available only for *N* = 318) but not AgeAccelGrim. There was a positive correlation between psychiatric and somatic burden of disease (spearman correlation r_s_ = 0.28). When adding the burden of psychiatric disease to the full model, somatic disease score as well as burden of psychiatric disease were non-significant (final reduced *N* = 299 available for this analysis) but the direction of association for burden of psychiatric disease remained the same, and was significant by itself in this subsample (*p* = 0.001 for AgeAccelGrim). The consistent directions of effects are depicted for DunedinPoAm and AgeAccelGrim with stratification by somatic disease status (non = 85, low=154, high = 103, Fig. S8).

**Table 2:** Result of multiple linear regressions of DNA methylation clocks with burden of psychiatric disease.

Burden of psychiatric disease					
Predicted variable	estimate	SE	*t* value	*p* value	adj. *p* value
AgeAccelHorvath	−0.050	0.0420	−1.194	0.233	0.933
AgeAccelHannum	−0.064	−0.0410	−1.571	0.117	0.468
AgeAccelPheno	−0.040	0.0570	−0.706	0.481	1.000
AgeAccelGrim	**0.155**	**0.0323**	**4.810**	**0.000002**	**0.000009**
DunedinPoAm	**0.002**	**0.0005**	**3.195**	**0.0015**	**0.006**

The models were controlled for age, sex, BMI, study, ethnicity, smoking status and cell proportions of CD8T, CD4T, NK, B lymphocytes and Monocytes except if covariate was used for the construction of specific clocks (in AgeAccelGrim sex and smoking status and in DunedinPoAm BMI were omitted). Significant effects are shown in bold and were adjusted for multiple testing. SE standard error.

To investigate whether the same association would be present, if only one disease was investigated, we repeated the analysis for the most common diagnosis in our sample, namely major depression (including all severities and coded as present versus not-present). In a multiple linear regression, we found no significant association of DunedinPoAm with both, current diagnosis (within the last month, *p* = 0.07, *N* = 149 with vs. *N* = 222 without) and lifetime diagnosis of major depression (over a month ago, *p* = 0.169, *N* = 63 with vs. *N* = 308 without). Similarly, associations with AgeAccelGrim were not significant in this analysis (current diagnosis: *p* = 0.082, *N* = 146 with vs. *N* = 218 without; lifetime diagnosis: *p* = 0.415, *N* = 60 with vs. *N* = 304 without).

### Interplay of childhood maltreatment and lifetime stress with burden of psychiatric disease and DunedinPoAm

**Fig. 3:**
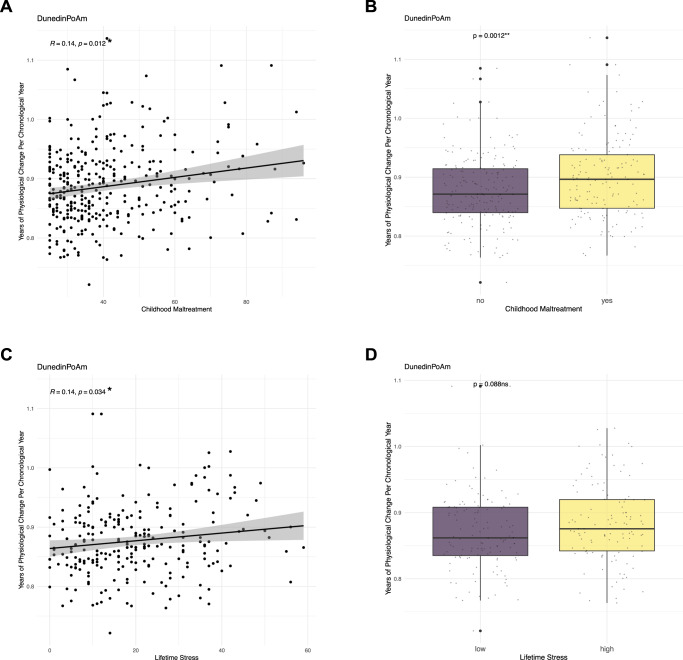
Associations between DunedinPoAm and childhood maltreatment or lifetime stress. Spearman correlation between DunedinPoAm (years of physiological change per chronological year) (**A**, **C**) on the y-axis and childhood maltreatment measured by CTQ score or cumulative lifetime stress measured by MEL score on the x-axis (Spearman correlation coefficient, r_s_). **B** displays a box plot and a *p*-value from a *t*-test of dichotomized childhood maltreatment status – abused (*N* = 149) vs. not abused (*N* = 191). The status abused was given if participant had moderate or severe abuse in any of the subscales of the questionnaire. Data was missing for 80 participants. **D** displays a box plot and a *p*-value from a *t*-test of dichotomized cumulative lifetime stress status – low (*N* = 128) vs. high (*N* = 115) lifetime stress categorized by the median score of the participants (median=18). This questionnaire was only available in the BeCOME cohort and was missing for 56 participants. ****P ≤ 0.0001; ***p ≤ .001; **p ≤ .01, *p ≤ .05. ns not significant.

We next investigated, whether childhood maltreatment (CM) and lifetime stress (LS) might influence the associations of burden of psychiatric disease with these two clocks, given that CM (total score measured by CTQ) and LS (total score measured by MEL) were both significantly associated with burden of psychiatric disease (β = 0.034, SE = 0.004, *t* = 7.796, *p* = 1.12 × 10^−13^, R^2^-adj. = 0.270 and β = 0.035, SE = 0.008, *t* = 4.422, *p* = 1.58 × 10^−05^, R^2^-adj. = 0.101 respectively) accounting for age, sex, ethnicity and study cohort (LS was only available in one cohort, details in the methods section). When analyzing association with the two clocks, CM but not LS was associated with DunedinPoAm when correcting for age, sex, ethnicity, smoking status, proportion of cell types and study cohort (β = 0.0004, SE = 0.0002, *t* = 1.987, *p* = 0.047, R^2^-adj. = 0.32, Fig. 3), while no significant associations were found with AgeAccelGrim. When including burden of psychiatric disease in the model, neither CM nor burden of psychiatric disease nor their interaction term remained significant predictors of DunedinPoAm (β = 0.0001, SE = 0.0002, *t* = 0.552, *p* = 0.58), which may relate to a reduction in sample size (*N* = 301). We then performed an interaction analysis focusing on more severe forms of maltreatment: moderate to severe physical and/or sexual abuse (exposed vs. non-exposed, using combined information from CTQ and M-CIDI) with burden of psychiatric disease on DunedinPoAm. The presence of moderate to severe physical abuse (but not sexual abuse) interacted with burden of psychiatric disease with nominal significance (but not withstanding correction for multiple testing) to increase the association with the pace of aging measured with DunedinPoAm (β = 0.005, SE = 0.002, *t* = 2.340, *p* = 0.02, adj.p = 0.08 Table S3). The association of DunedinPoAm with burden of psychiatric disease and CM was not influenced by school education and household income (Fig. S9). In fact, burden of psychiatric disease showed a steeper slope of association with faster pace of aging in the 36 individuals exposed to physical abuse (Fig.S10).

## Discussion

In this study, biological aging (BA) was quantified with five different measurements of epigenetic age in patients with psychiatric disorders and healthy controls. Subjects with a higher burden of psychiatric disease, a cumulative diagnostic score of psychiatric disorders across lifetime, presented with higher AgeAccel as measured by DNAm GrimAge and faster pace of BA as measured by DunedinPoAm. Differences in epigenetic age acceleration remained significant after accounting for age, sex, ethnicity, BMI, smoking status, cell type proportions and investigated cohort. This association was not driven by differences in socioeconomic status, psychiatric medication or somatic disease alone. The association of burden of psychiatric disease with the pace of aging (DunedinPoAm) was further accelerated in individuals exposed to childhood physical abuse. Importantly, the association was absent when only including the presence or absence of the most common single psychiatric diagnosis in the sample, major depression. Overall, these findings suggest that an increased transdiagnostic burden of psychiatric disease is associated with epigenetic age acceleration in the DNAm GrimAge clock and faster pace of aging (DunedinPoAm), the latter being exacerbated by additional exposure to physical abuse.

Our findings point to the cumulative risk conferred by comorbid psychiatric disorder on BA and thus age-associated disease. Interestingly, associations are observed even within the spectrum of mood and anxiety disorders. Stronger associations with age-associated diseases and reduced life expectance have been reported for other psychiatric disorders (e.g. schizophrenia, [5]). Our lack of association with the diagnosis of depression alone differs from early findings [28], but possibly due to heterogeneity in previous studies investigating single diagnoses that may not have mapped all lifetime comorbidity. Since somatic disease was only available in a subset of individuals and was intercorrelated with burden of psychiatric disease we were not able to disentangle individual effects.

In addition to psychiatric diseases themselves, major risk factors, such as childhood maltreatment (CM) or chronic psychological stress (lifetime stress, LS) also have been associated with lasting changes in biological systems and shown to negatively influence age-related diseases [65]. Meta-analytic evidence of leukocyte telomere length shortening, a different measure of aging, described a relation with CM [66, 67] and several psychiatric disorders [68–70]. As for epigenetic age estimates, associations of psychological stress with epigenetic age acceleration have been reported [22, 34, 71]. Furthermore, faster pace of aging (calculated with the older 2015 version) was found in subjects with higher adverse childhood experiences [72] but others did not find significant association of epigenetic aging with CM [22, 34, 36].

Our analysis showed not only the expected association of CM with the burden of psychiatric disease, but also with the pace of aging (DunedinPoAm). In fact, we observed an interaction of burden of psychiatric disease with exposure to physical abuse, with exposed subjects having an even higher pace of aging with increased burden of disease. Although not surviving Bonferroni correction, these associations hint towards epigenetic mechanisms that influence both, the process of aging and development of psychiatric stress-related disease, reflecting a joint influence on faster pace of aging. In fact, a history of CM was shown to lead to an earlier age at onset, great symptom severity and comorbidity and poorer treatment outcome [73]. Teicher et al. argue that individuals with CM may represent a transdiagnostic subtype and should be used for stratification of patients with psychiatric disorders [73]. CM, therefore, may also represent a transdiagnostic risk factor influencing BA, but these findings need replication in larger cohorts.

The lack of association with LS, while supporting the theory describing the importance of developmental time-point for the impact of environmental stress states on epigenetics [74], could also be related to lack of power or complex confounding of these assessments.

As expected, associations were not observed in all investigated DNAm clocks. To date, it is unknown whether different clocks indeed quantify different facets of the aging process [10]. Our findings are in line with previously reported low agreement among different measures of BA (r = 0.3–0.5, even lower between pace of aging and chronological clocks [10, 75] (Fig. S6). Different DNAm clocks, as previously stressed [12], do not share many CpGs (Fig. S7), supporting the hypothesis that different estimators capture different underlying biological mechanisms of aging and age-related diseases. Therefore, estimators might be more or less useful as biomarkers for a specific disease phenotype [11]. For example, DunedinPoAm possibly captures biological processes associated with CM (specifically physical abuse) better than others. Associations also might be specific to a single epigenetic clock [76] or a specific type of CM [33].

When considering our results, several important limitations should be acknowledged. Our cohort included mainly Caucasians, that also presented with several additional factors that have been shown to possibly positively influence BA [77]. The majority of the subjects had at least 12 years of high school education (67,1%) and had a mean BMI of 24.6 (kg/m^2^). Only very few of the participants were current smokers and close to 40% never smoked. Accordingly, we found generally favorable BA in our cohort (Table S4). Furthermore, some diagnoses are underrepresented in our analysis (Fig. S2). Therefore, an investigation in a larger, ethnically, diagnostically and socioeconomically more heterogenous population will be important before generalizing our findings. Despite considering many covariates in the analysis (individual effect sized delineated in Table S5), there might be other unknown covariates driving the association including physical activity [60, 78] or dietary habits [60]. Moreover, measurements of CM and LS were collected retrospectively in adulthood, and might reflect different information than prospective forms of assessment [79]. Furthermore, our maltreatment exposed group was rather small and the interaction analysis will need replication. Finally, we performed a cross-sectional analysis so we cannot identify causal relationships or determine the direction of effect. Due to the cross-sectional nature of our data as well as concerns of temporal distinction and of reverse causation, we did not perform a mediation analysis to explore the relationships between CM, burden of psychiatric disease and somatic disease on biological aging. This important topic needs to be explored in future longitudinal studies.

Our strongest and most consistent associations were observed with DunedinPoAm. It represents the ‘rate’ of BA and is scaled with ‘years of physiological change per chronological year’ rather than units of years [17]. Although our results are preliminary and explorative in nature, they represent the first investigation of DunedinPoAm in a larger clinical psychiatric population. Epigenetic clocks as biomarkers, integrate multiple, partly correlated, features, that contribute to biological aging, and although the independent relative variance explained by burden of psychiatric disease is low (R^2^ = 0.035), individuals with higher burden of psychiatric disease would be at higher risk for accelerated biological aging. Increased pace of aging has been associated with increased morbidity and this biomarker could thus provide information on which patient may benefit most from interventions for modifiable risk factors [80]. Given the dramatically shortened life expectancy of psychiatric patients, improving detection and management of psychiatric patients at risk for age-associated diseases, already at young age, can contribute to personalized medicine and reduction of mortality. For this, evaluation of change in BA after interventions such as antidepressant medication or running therapy would be important, to evaluate their actual biological impact. This is currently attempted in a prospective randomized trial in MDD and anxiety disorders [81]. As such, measure of BA may serve as important biomarker to improve life expectancy in psychiatric patients.

## Supplementary information

Supplementary Information
