# Supplementary material for: Transdiagnostic evaluation of epigenetic age acceleration and burden of psychiatric disorders
Source: Neuropsychopharmacology. 2023 Apr 17;48(9):1409–17. doi: 10.1038/s41386-023-01579-3 (PMC10354057; doi:10.1038/s41386-023-01579-3)
Supplement: Supplementary file 1 — Supplementary Information [file 41386_2023_1579_MOESM1_ESM.pdf]

Supplementary Information

for the article entitled "Transdiagnostic evaluation of epigenetic age acceleration and burden of psychiatric disorders", Yusupov et al.

Summary

Table of Contents

1. Supplementary Methods:

1.1 Child Trauma Questionnaire (CTQ)

1.2 Munich Event-Questionnaire (MEL)

1.3 Somatic disease score

1.4 List of biomarkers used in the construction of the PhenoAge, GrimAge and DunedinPoAm algorithms

1.5 Sensitivity Analysis: Median absolute error

2. Supplementary Figures:

Figure S1. Distribution of burden of psychiatric disease

Figure S2. Broad diagnostic categories of the cohort

Figure S3. A. Distributions of DNA methylation age acceleration, AgeAccel, (years) measured by different clocks. B. Distribution of DunedinPoAm (years of physiological change per chronological year)

Figure S4. Correlation matrix of computed cell type proportions

Figure S5. Correlations of DNA methylation age (years) calculated by different clocks (A-D) with age (years)

Figure S6. Correlations of DunedinPoAm with DNA methylation age acceleration (AgeAccel) measured by different clocks

Figure S7. Distinct intersections of CpGs shared by different DNA methylation clocks

Figure S8. Stratification of biological age by somatic disease score and status of burden of psychiatric disease

Figure S9. Stratification of biological age measured with DunedinPoAm by school education/household income and abuse status as well as burden of psychiatric disease

Figure S10. Exposure to physical abuse in childhood interacts with burden of psychiatric disease to increase the pace of aging

3. Supplementary Tables:

Table S1. Post hoc linear regression results stratified by sex for the DunedinPoAm model

Table S2. Post hoc linear regression results stratified by sex for the AgeAccelGrim model

Table S3. Results of linear regression for the interaction between physical abuse and burden of psychiatric disease on DunedinPoAm

Table S4. Descriptive statistics of DNA methylation age accelerations of the different DNA methylation clocks

Table S5. Results of linear regression for the DunedinPoAm model

4. References

1. Supplementary Methods

1.1 Child Trauma Questionnaire (CTQ)

CTQ is a 28-item retrospective assessment of maltreatment until the age of 18 (1, 2). Items are rated on a 5-point Likert scale from never to very often. Five subscales can be generated (emotional abuse, physical abuse, sexual abuse, emotional neglect, physical neglect) including five items each (ranging from 5 to 25 points each). A total score was calculated by adding the points of the subscales (max. 125 points).

1.2 Munich Event-Questionnaire (MEL)

MEL is a retrospective self-report based on the Munich Event List, which contains positive and negative events throughout life (3, 4). The following significant events from different domains composed a total score of potentially stressful events over the course of life:

A. Relationship (6 items): Death of partner, separation from partner, fight with partner, fight with partners' family, change partners employment, divorce.

B. Disease/Lost (8 items): Severe disease of partner, death of family member, pregnancy, sexual difficulties, addition to family, death of close friend, children moved out, severe disease

C. Change in Residency/Work/Finance (9 items): Significant change in income, significant borrowing, change of residence, loss of work, occupational change, commercial change, job change, abandonment education, imprisonment.

D. Change in healthy balance (4 items): Retirement, abandonment of hobbies, significant change in work conditions, problems with boss.

Each of the events above (27 items) was rated with a 4-point Likert scale (0 = never, 1 = once, 2 = twice, 3 = multiple times). Cumulative lifetime stress exposure was indicated by the total number of events and their frequencies.

1.3 Somatic disease

For the assessment of somatic and metabolic diseases among psychiatric patients the self-developed questionnaire of the Max Planck Institute of Psychiatry was used. Questions (yes/no) from different domains were stated as follows: "Has a doctor or healthcare professional ever told you that you have any of the following medical conditions":

A. Metabolic: Diabetes/high levels of blood sugar/need of blood sugar controls, birth of a heavy child (>4100 g), overweight, gout/high uric acid, high levels of blood lipids.

B. Cardiovascular/Respiratory: Circulatory disturbances of legs, wound healing disturbances of feet, slower walk due to pain, breathlessness while walking, high blood pressure, prior cardiac infarction, prior stroke, kidney malfunction, chronic bronchitis.

C. Immunological: Asthma, allergies/hay fever, psoriasis, neurodermatitis.

D. Other: Osteoporosis, arthrosis, rheumatism, migraine, past cancer, further additional not mentioned in the questionnaire.

The somatic disease score was constructed by addition of items reported by the subjects concerning general pathological metabolic conditions and other somatic diseases.

1.4 List of biomarkers used in the construction of the PhenoAge, GrimAge and DunedinPoAm algorithms

In addition to age, the following 9 biomarkers were used to construct PhenoAge (5): C-reactive protein, Albumin, Creatinine, Alkaline phosphatase, Glucose, Lymphocyte percent, Mean (red) blood cell volume, Red blood cell distribution width, White blood cell count.

Besides age, sex and C-reactive protein, additional DNAm based biomarker surrogates were used to construct GrimAge (6): smoking pack-years, plasminogen activation inhibitor 1 (PAI-1), growth differentiation factor 15 (GDF-15), beta-2 macroglobulin (B2M), Adrenomedullin levels (ADM), Cystatin C, Leptin and tissue inhibitor metalloproteinase 1 (TIMP-1).
The following 18 biomarkers were longitudinally evaluated by Belsky et al. to construct the Pace of Aging (7) and its later modification, DunedinPoAm (Dunedin Pace of Aging Methylation) (8): Glycated hemoglobin, Forced expiratory volume in one second (FEV1), Blood pressure (mean arterial, pressure), Total cholesterol, C-reactive protein, Creatinine clearance, Urea nitrogen, Cardiorespiratory fitness (VO2Max), Waist-hip ratio, Forced vital capacity ratio (FEV1/FVC), Body mass index (BMI), Leukocyte telomere length (LTL), Lipoprotein(a), Triglycerides, Periodontal disease, White blood cell count, High density lipoprotein (HDL), and Apolipoprotein B100/A1 ratio. For further details about construction of the clocks are available at the original publications (7, 8).

1.5 Sensitivity analysis: Median absolute error

As suggested by Horvath et al. to exclude poor calibration of DNAm age estimates (9), we have calculated the median absolute difference between the DNA methylation age and chronological age, also referred to as the median “error”, for the different DNA methylation age estimators in our cohort. The mean absolute error (in years) was for the following DNA methylation clocks: Horvath: 4.443, Hannum: 4.403, PhenoAge: 6.204, GrimAge: 3.768.

2. Supplementary Figures:

Figure S1. Distribution of burden of psychiatric disease. The following disorders were evaluated with the computer-based slightly modified version of the Munich-Composite International Diagnostic Interview (DIA-X/M-CIDI)(10) and included in the score: depressive disorders, dysthymia, mania and bipolar disorder, anxiety disorders - panic attacks, panic disorder, agoraphobia, specific and social phobias, generalized anxiety disorder, post-traumatic stress disorder, obsessive-compulsive disorders, nicotine/caffeine/alcohol/illegal substances use and dependency. The distribution was stratified by both cohorts and included in the main analysis.

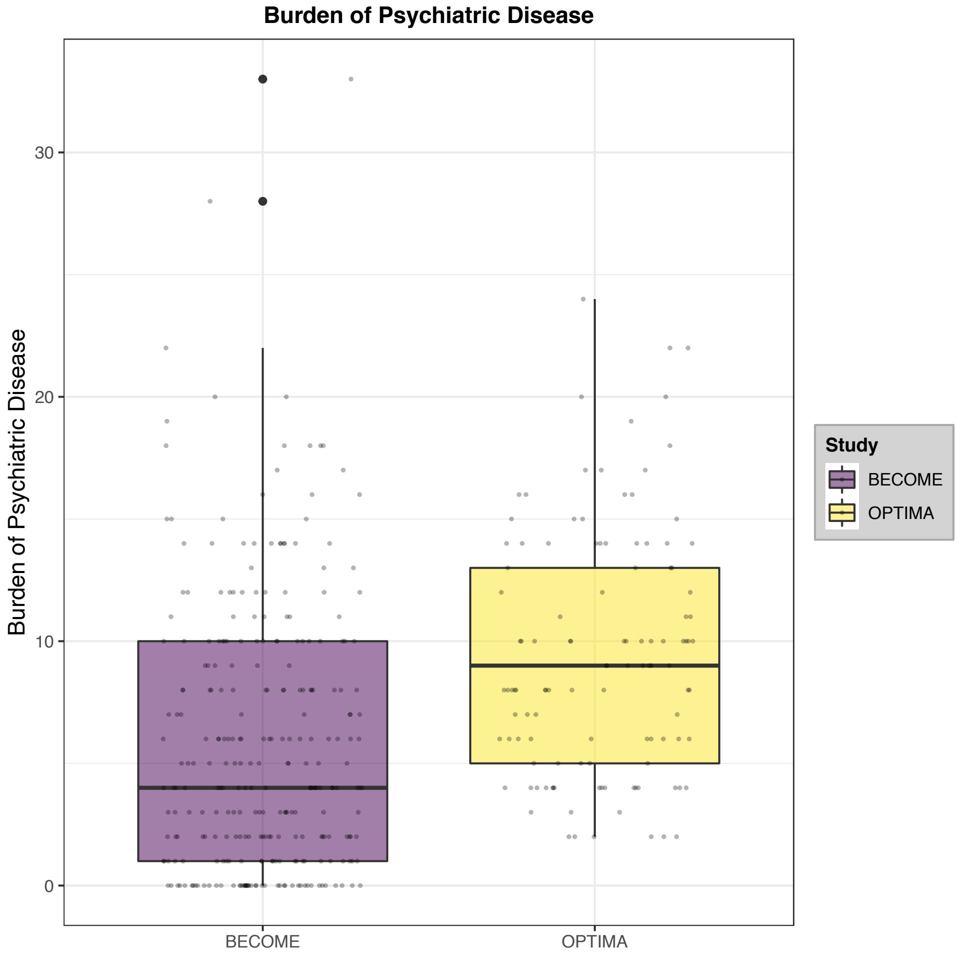

Figure S2. Broad diagnostic categories of the cohort. Displayed in percent and stratified by occurrence: (A) current (in the last month) and (B) lifetime (>1 month). Included in the category depression were depressive episodes and recurrent depressive disorders of various severities (mild, moderate or severe with/without psychotic symptoms). Included in the category bipolar were includes bipolar affective disorders with episodes of different types (hypomanic, manic with/without psychotic symptoms, depressive or mixed).

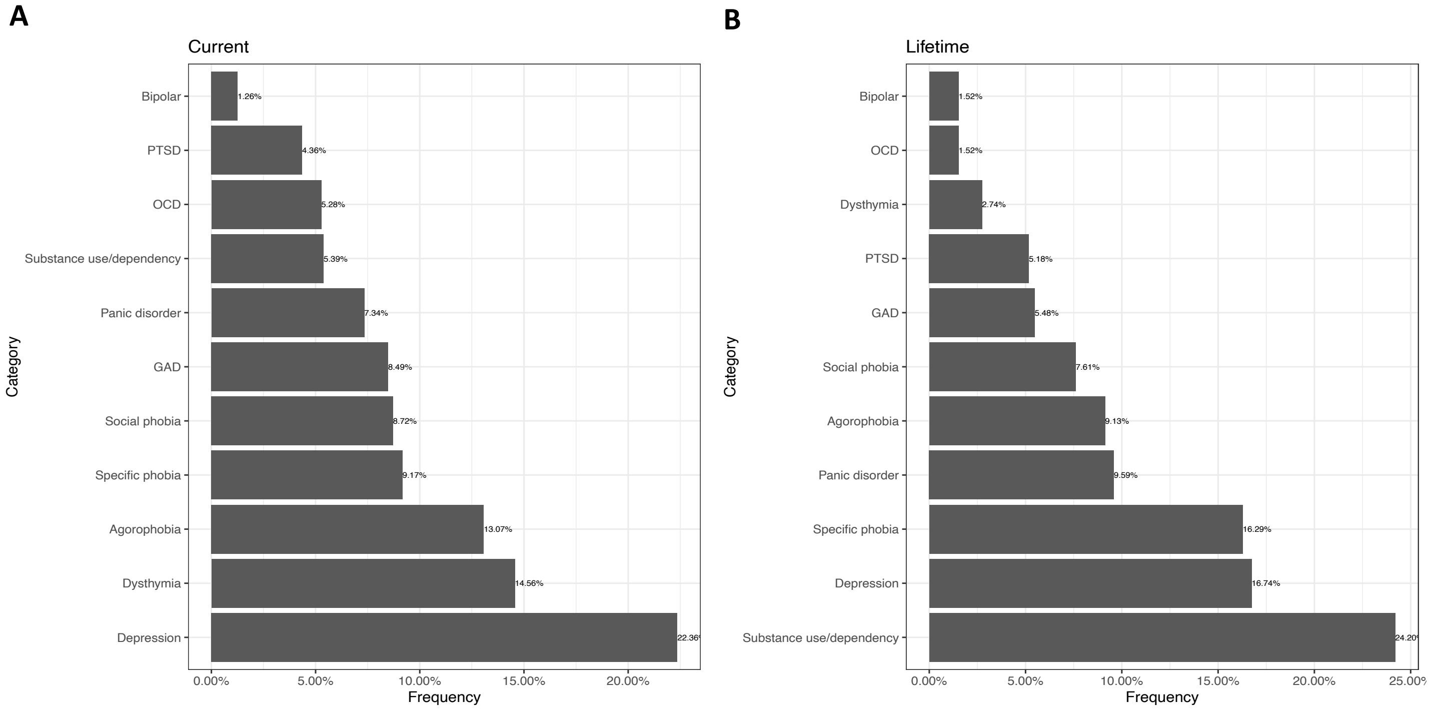

Figure S3: A. Distributions of DNA methylation age acceleration (years) measured by different clocks. B. Distributions of DunedinPoAm (years of physiological change per chronological year)

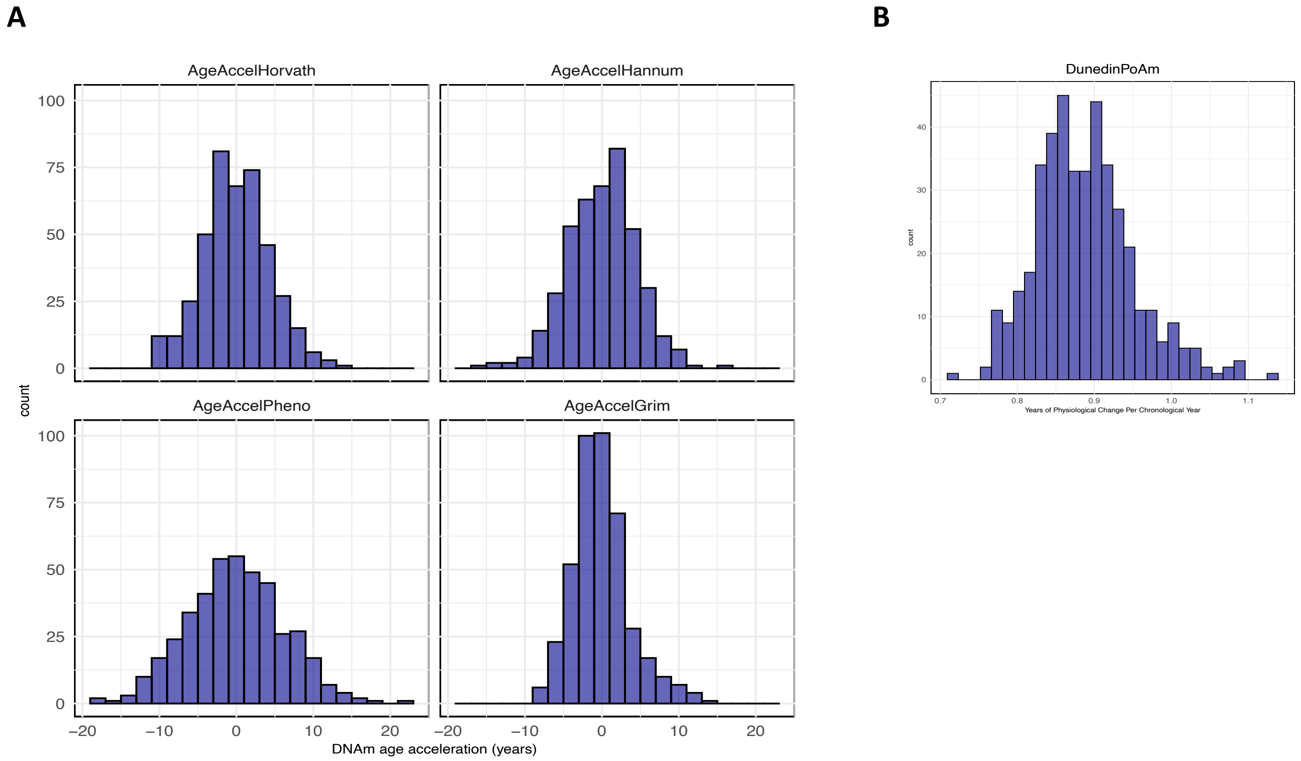

Figure S4. Correlation matrix of computed cell type proportions. Persons' correlation coefficients are presented. Cell type proportions were calculated using the advanced analysis option in Horvaths' New Methylation Age Calculator (https://dnamage.genetics.ucla.edu/new) according to Houseman et al. (9, 11, 12).

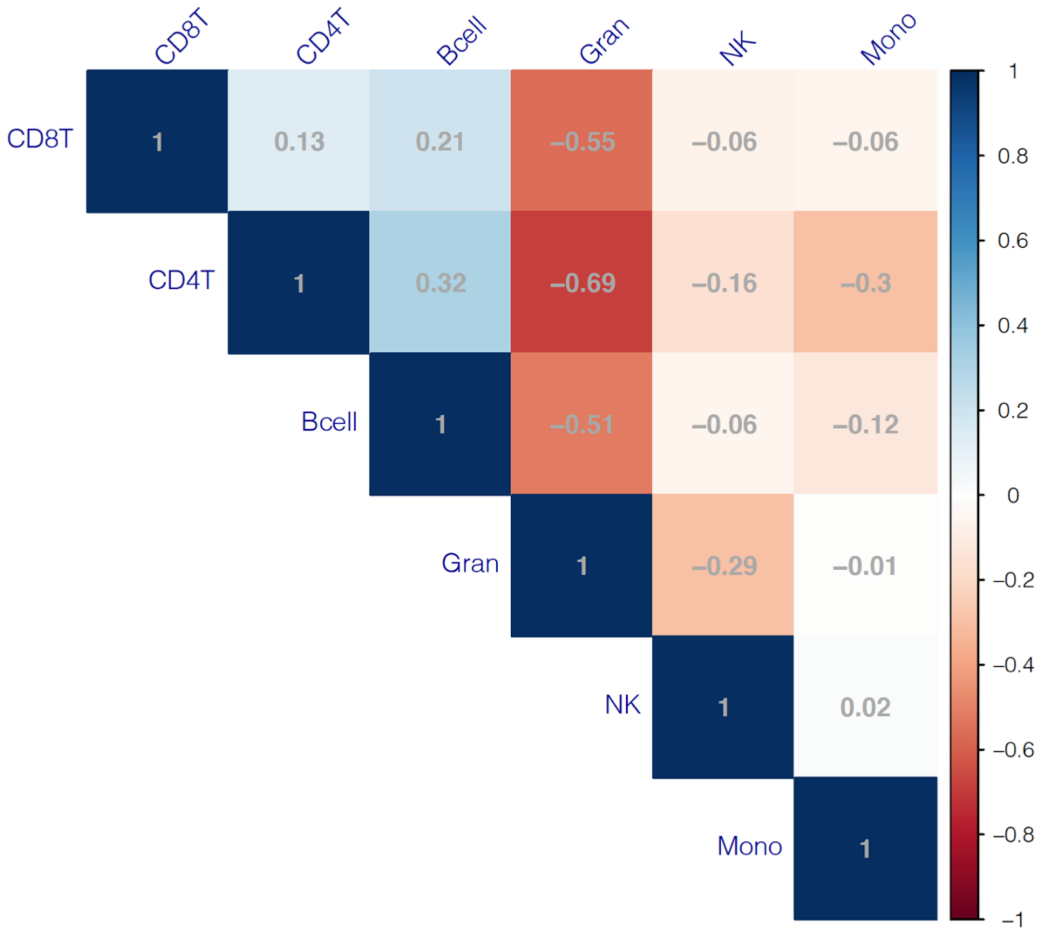

Figure S5. Correlations of DNA methylation age (years) calculated by different clocks (A-D) with age (years). Persons' correlation coefficients are presented.

**** P ≤ 0.0001; *** = p ≤ .001; ** p ≤ .01; * = p ≤ .05.; ns = not significant.

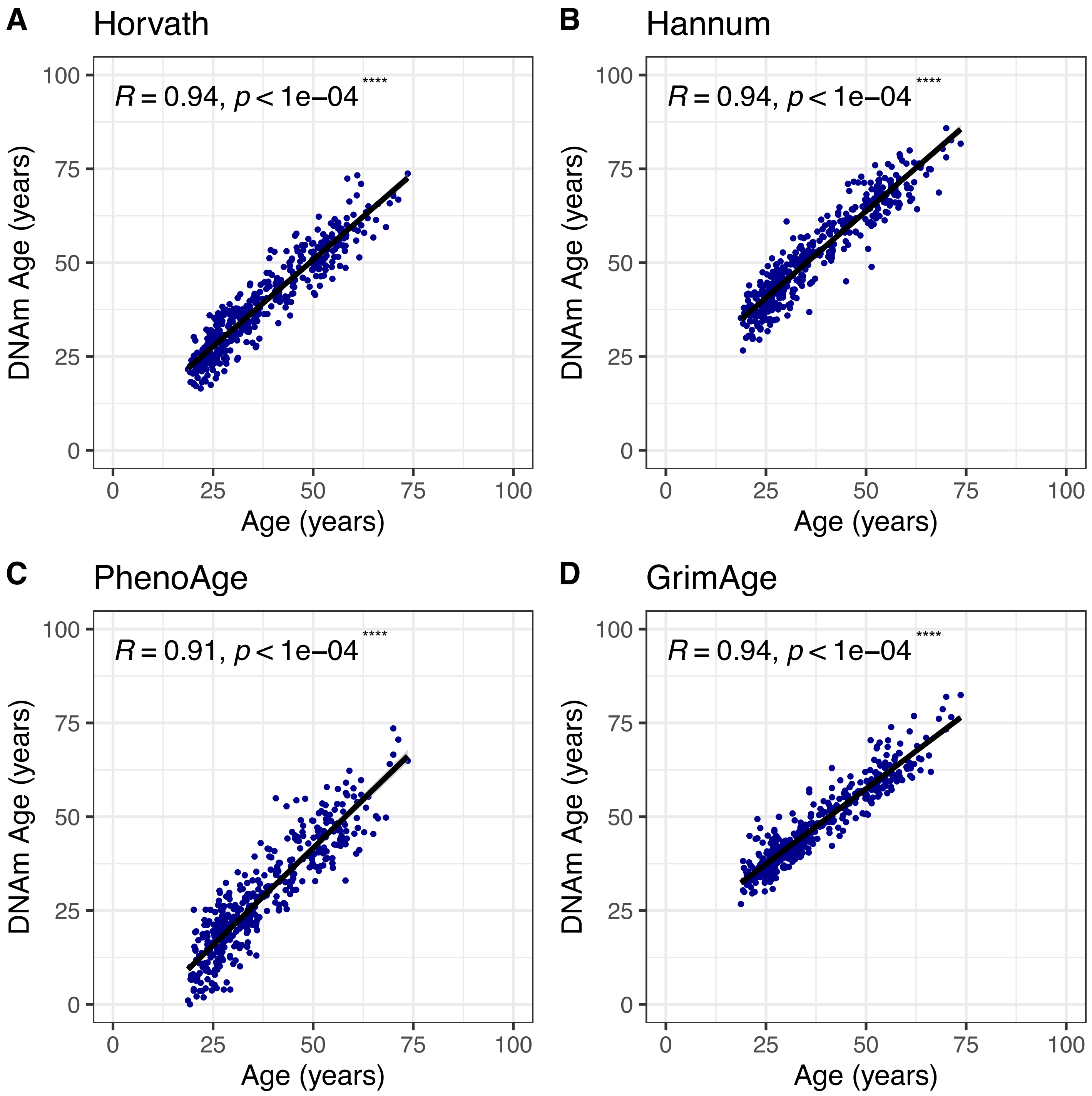

Figure S6. Correlations of DunedinPoAm with DNA methylation age acceleration (AgeAccel) measured by different clocks. Persons' correlation coefficients are presented. **** P ≤ 0.0001; *** = p ≤ .001; ** p ≤ .01; * = p ≤ .05.; ns = not significant.

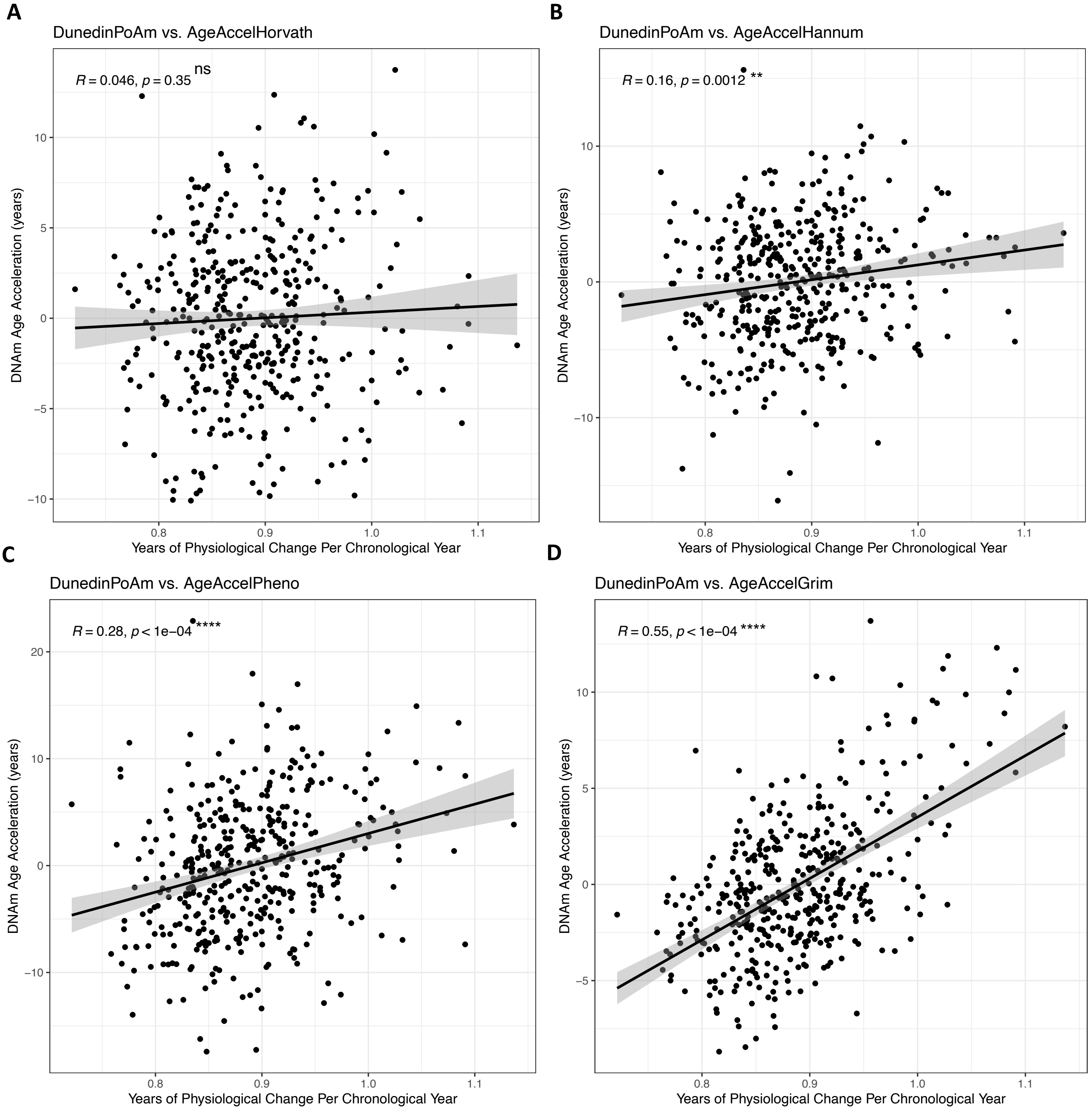

Figure S7. Distinct intersections of CpGs shared by different DNA methylation clocks. The number of CpGs per Clock is displayed on the left side. Distinct CpG intersections are displayed as an UpSet plot on the left side. A single point represents CpGs present only in the specific set, whereas two or more points linked by a line represent distinct shared CpGs between sets. Matrix-based visualization was performed using the *UpSetR* R package, version 1.4.0 (13).

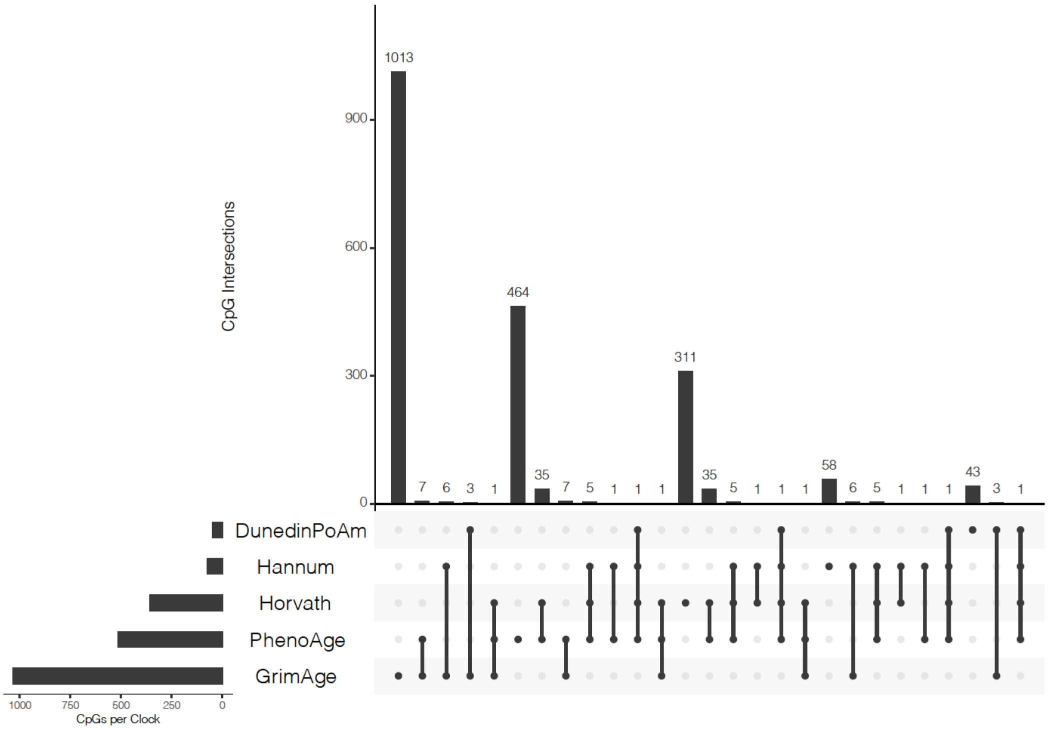

Figure S8. Stratification of biological age by somatic disease score and status of burden of psychiatric disease. Stratification is shown for DunedinPoAm (A) and AgeAccelGrim (B). Kruskal–Wallis test was performed between groups of somatic disease status (none = 85, low = 154, high = 103) according to status of burden of psychiatric disease (control with no diagnosis = 47, low = 185 and high = 163), both categorized by the median score of participants with score > 0 (median = 2 and 7 respectively). Information about somatic disease was available for 342 subjects. p-values are shown.

A

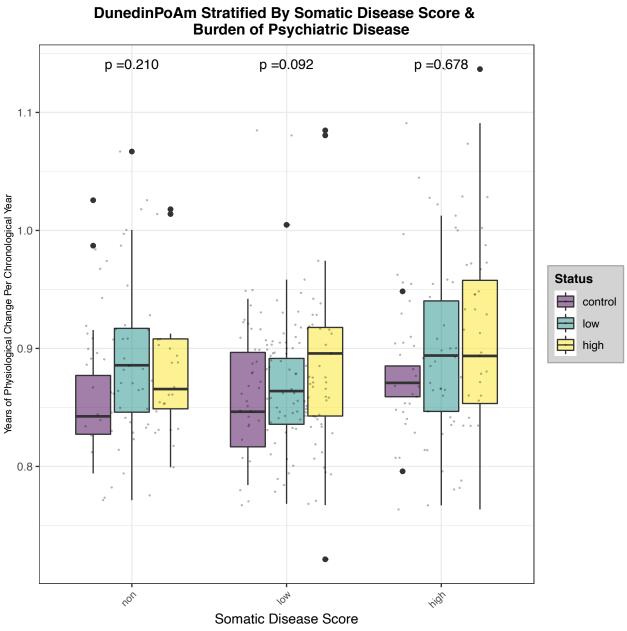

B

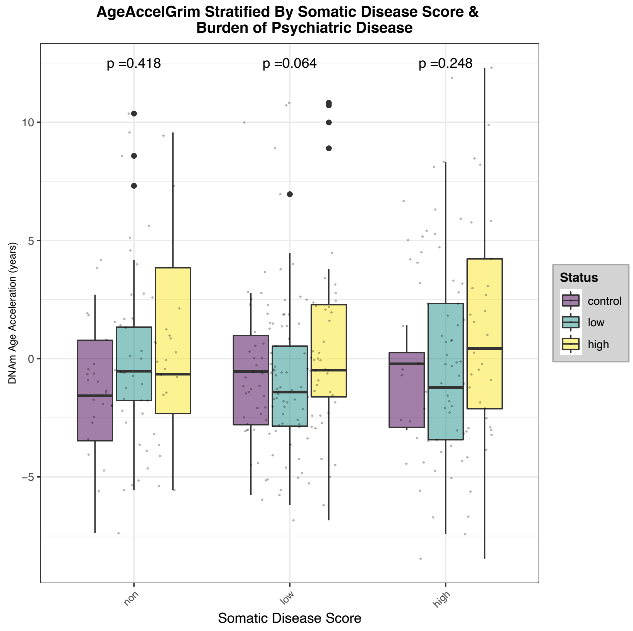

Figure S9. Stratification of biological age measured with DunedinPoAm by school education/household income and abuse status as well as burden of psychiatric disease. Wilcoxon signed-rank test was performed between groups of school education (A) and household income (B) according to abuse status (defined as abused if a moderate or severe abuse present in any subscale). Kruskal–Wallis test was performed between groups of school education (C) and household income (D) according to status of burden of psychiatric disease (control with no diagnosis = 47, low = 185 and high = 163) categorized by the median score of participants with score > 0 (median = 7). Information was available for 378 (school education) and 202 (household income) subjects. Subjects with no school graduation (N = 3) and still in school (N = 1) were omitted for this analysis. p-values are shown.

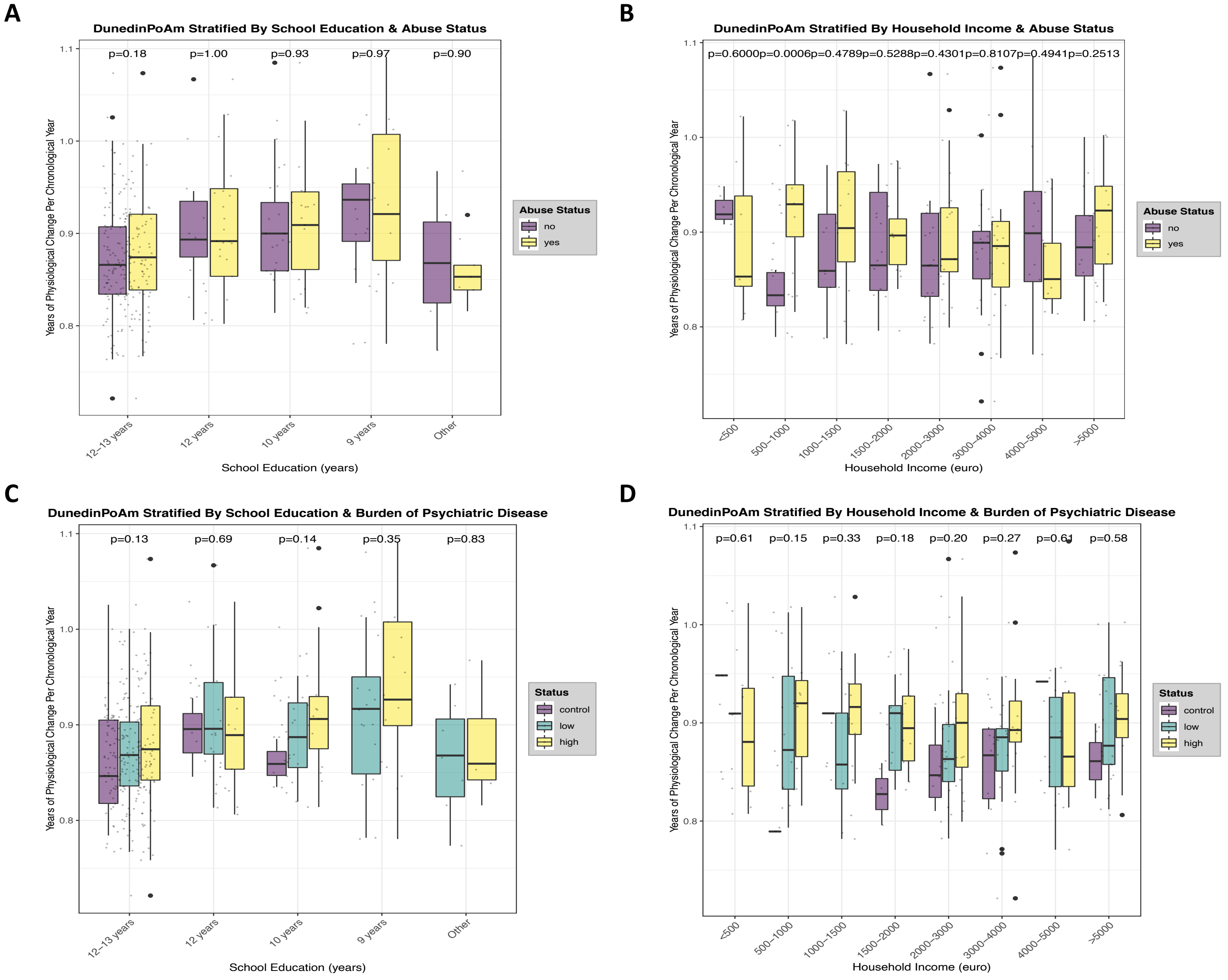

Figure S10. Exposure to physical abuse in childhood interacts with burden of psychiatric disease to increase the pace of aging. Linear associations and beta coefficients for both groups are shown. Data was available for 391 subjects (exposed = 36, non-exposed = 355).

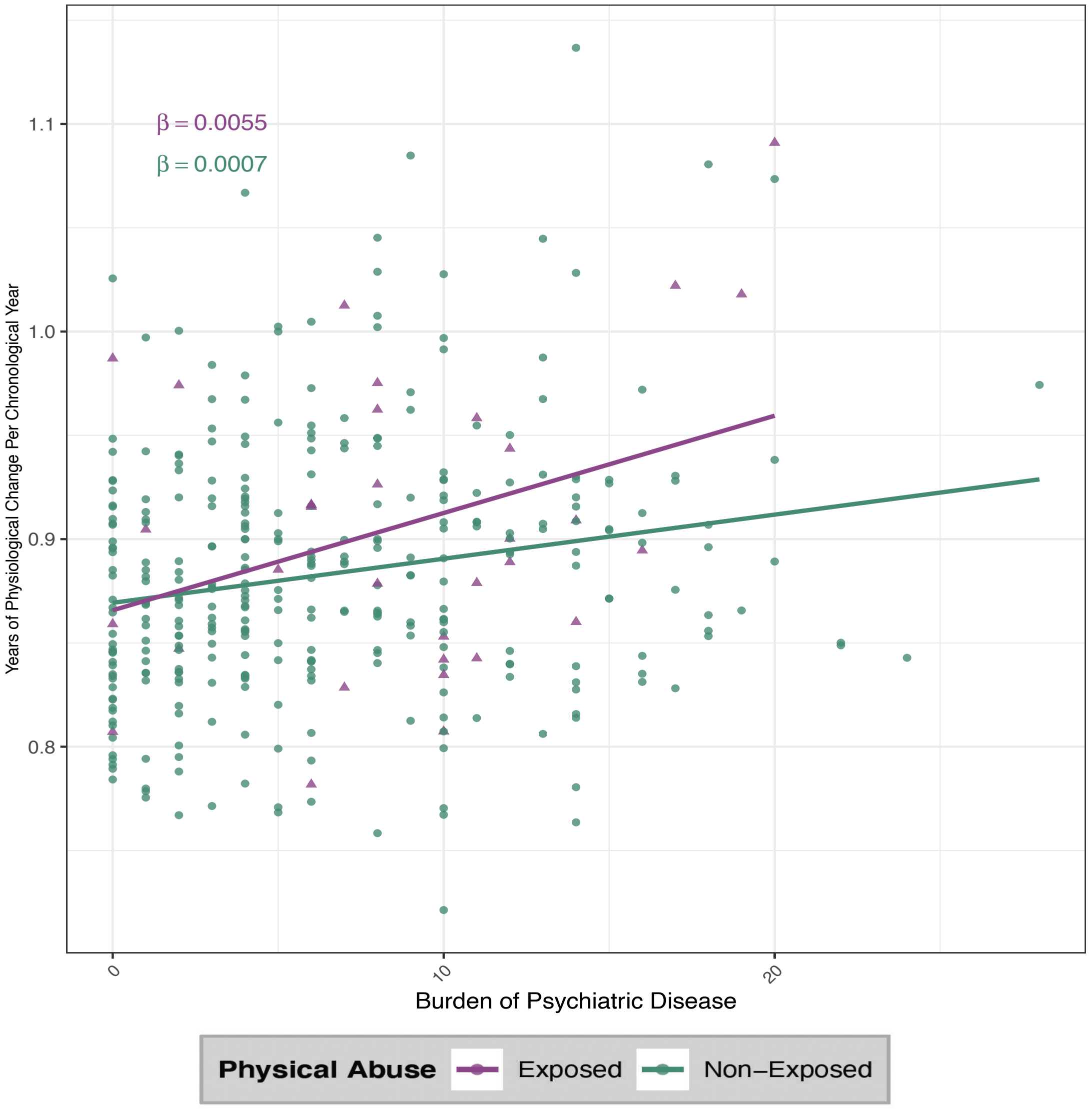

3. Supplementary Tables

Table S1. Post hoc linear regression results stratified by sex for the DunedinPoAm model. The model was controlled for age, study, population stratification (two first principal components), smoking status and cell proportions of CD8T, CD4T, NK, B lymphocytes and Monocytes. SE = standard error.

	**Females (N = 223)**				**Males (N = 148)**			
**Variable**	**estimate**	**SE**	**t value**	**p value**	**estimate**	**SE**	**t value**	**p value**
Age	0.0001	0.0003	0.215	0.830	0.0013	0.0004	2.817	0.006
Study [OPTIMA]	0.0133	0.0082	1.619	0.107	0.0157	0.0110	1.432	0.154
PC1	-0.6058	0.4723	-1.283	0.201	-0.7493	0.5613	-1.335	0.184
PC2	0.1105	0.5122	0.216	0.829	0.9495	0.7300	1.301	0.195
Smoking Status [Former Smoker]	-0.1575	0.0513	-3.073	0.002	-0.1478	0.0404	-3.650	0.0004
Smoking Status [Never Smoker]	-0.1676	0.0512	-3.270	0.001	-0.1589	0.0425	-3.736	0.0003
CD8T	-0.5579	0.1084	-5.147	6.08×10^{-5}	-0.3808	0.1442	-2.641	0.009
CD4T	-0.2500	0.0736	-3.398	0.001	-0.3004	0.1007	-2.984	0.003
B lymphocytes	-0.4440	0.1685	-2.634	0.009	0.3090	0.2635	1.173	0.243
NK cells	-0.1486	0.1097	-1.354	0.177	-0.3181	0.1246	-2.554	0.012
Monocytes	0.2552	0.1810	1.410	0.160	-0.0759	0.2413	-0.314	0.754
Burden of psychiatric disease	0.0020	0.0007	2.756	0.006	0.0015	0.0008	1.962	0.052
R^2 (adj. R^2)	0.379 (0.343)				0.381 (0.326)			

Table S2. Post hoc linear regression results stratified by sex for the AgeAccelGrim model. The model was controlled for BMI, study, population stratification (two first principal components) and cell proportions of CD8T, CD4T, NK, B lymphocytes and Monocytes. SE = standard error.

	**Females (N = 220)**				**Males (N = 144)**			
**Variable**	**estimate**	**SE**	**t value**	**p value**	**estimate**	**SE**	**t value**	**p value**
BMI	0.0638	0.0383	1.668	0.097	0.0357	0.0691	0.517	0.606
Study [OPTIMA]	0.4937	0.4752	1.039	0.300	2.3035	0.6859	3.358	0.001
PC1	-36.9135	27.9159	-1.322	0.187	-9.9514	36.0735	-0.276	0.783
PC2	3.0400	30.1187	0.101	0.920	31.7588	46.2507	0.687	0.493
CD8T	-8.0540	6.1557	-1.308	0.192	-10.6150	8.3525	-1.271	0.206
CD4T	-12.5586	4.3194	-2.908	0.004	-14.3718	6.3594	-2.260	0.025
B lymphocytes	-18.0835	9.8866	-1.829	0.069	12.6343	16.3491	0.773	0.441
NK cells	-19.7974	6.2117	-3.187	0.002	-21.8654	7.8765	-2.776	0.006
Monocytes	-1.7773	10.7276	-0.166	0.869	15.4883	15.2513	1.016	0.312
Burden of psychiatric disease	0.1768	0.0419	4.216	3.71×10^{-5}	0.1051	0.0490	2.143	0.034
R^2 (adj. R^2)	0.247 (0.211)				0.234 (0.177)			

Table S3. Results of linear regression for the interaction between physical abuse and burden of psychiatric disease on DunedinPoAm. The model was controlled for age, sex, study, population stratification (two first principal components), smoking status and cell proportions of CD8T, CD4T, NK, B lymphocytes and Monocytes. Significant effects are shown in bold. SE = standard error.

**DunedinPoAm**				
**Variable**	**estimate**	**SE**	**t value**	**p value**
Age	0.0004	0.0003	1.509	0.132
Sex [Male]	-0.0027	0.0062	-0.434	0.665
**Study [OPTIMA]**	0.0191	0.0070	2.739	**0.006**
PC1	-0.6533	0.3608	-1.811	0.071
PC2	0.5427	0.4224	1.285	0.200
**Smoking Status [Former Smoker]**	-0.1616	0.0308	-5.253	**2.68×10^{-7}**
**Smoking Status [Never Smoker]**	-0.1743	0.0313	-5.576	**5.13×10^{-8}**
**CD8T**	-0.4965	0.0878	-5.657	**3.34×10^{-8}**
**CD4T**	-0.3056	0.0614	-4.977	**1.04×10^{-6}**
B lymphocytes	-0.2045	0.1463	-1.398	0.163
**NK cells**	-0.2598	0.0831	-3.125	**0.002**
Monocytes	0.0487	0.1460	0.333	0.739
Burden of psychiatric disease	0.0007	0.0006	1.324	0.186
Physical abuse [Exposed]	-0.0285	0.0210	-1.356	0.176
**Burden of psychiatric disease x Physical abuse [Exposed]**	0.0048	0.0020	2.340	**0.020**
R^2 (adj. R^2)	0.373 (0.345)			

Table S4. Descriptive statistics of DNA methylation age accelerations of the different DNA methylation clocks. Mean, standard deviation, median, maximum and minimum values in years as well as p value from a t-test are displayed for the chronological age and each of the DNA methylation age accelerations.

	BECOME (N=301)	OPTIMA (N=119)	P-value
**Age (years)**			
Mean (SD)	35.3 (12.1)	42.9 (13.5)	<0.001
Median [Min, Max]	31.8 [18.7, 66.2]	44.7 [19.2, 73.6]	
**AgeAccelHorvath (years)**			
Mean (SD)	0.130 (4.23)	-0.381 (4.77)	0.309
Median [Min, Max]	-0.0611 [-9.81, 12.4]	-0.426 [-10.1, 13.7]	
**AgeAccelHannum (years)**			
Mean (SD)	0.0287 (4.41)	0.0212 (4.43)	0.988
Median [Min, Max]	0.407 [-16.1, 15.6]	0.184 [-11.9, 11.5]	
**AgeAccelPheno (years)**			
Mean (SD)	-0.183 (5.99)	0.226 (6.88)	0.57
Median [Min, Max]	0.122 [-17.4, 17.0]	-0.400 [-17.2, 22.9]	
**AgeAccelGrim (years)**			
Mean (SD)	-0.605 (3.22)	1.29 (4.46)	<0.001
Median [Min, Max]	-0.796 [-8.69, 11.2]	0.755 [-8.01, 13.7]	
**DunedinPoAm**			
Mean (SD)	0.879 (0.0589)	0.912 (0.0689)	<0.001
Median [Min, Max]	0.871 [0.721, 1.09]	0.913 [0.782, 1.14]	

Table S5. Results of linear regression for the DunedinPoAm model. The model was controlled for age, sex, study, population stratification (two first principal components), smoking status and cell proportions of CD8T, CD4T, NK, B lymphocytes and Monocytes. Significant effects are shown in bold and were adjusted for multiple testing. SE = standard error.

**DunedinPoAm**				
**Variable**	**estimate**	**SE**	**t value**	**p value**
Age	0.0004	0.0002	1.597	0.444
Sex [Male]	-0.0013	0.0061	-0.207	3.344
**Study [OPTIMA]**	0.0167	0.0065	2.570	**0.042**
PC1	-0.6939	0.3571	-1.943	0.211
PC2	0.4696	0.4199	1.118	1.056
**Smoking Status [Former Smoker]**	-0.1561	0.0309	-5.057	**2.728×10^{-6}**
**Smoking Status [Never Smoker]**	-0.1676	0.0313	-5.348	**6.36×10^{-7}**
**CD8T**	-0.4988	0.0863	-5.783	**6.4×10^{-8}**
**CD4T**	-0.2820	0.0594	-4.747	**1.2×10^{-5}**
B lymphocytes	-0.2161	0.1410	-1.533	0.505
**NK cells**	-0.2420	0.0810	-2.993	**0.012**
Monocytes	0.0775	0.1436	0.540	2.359
**Burden of psychiatric disease**	0.0016	0.0005	3.195	**0.006**
R^2 (adj. R^2)	0.354 (0.330)			

4. References

1. Bernstein DP, Fink L, Handelsman L, Foote J, Lovejoy M, Wenzel K, et al. Initial reliability and validity of a new retrospective measure of child abuse and neglect. Am J Psychiatry. 1994;151(8):1132-6.
2. Bernstein DP, Stein JA, Newcomb MD, Walker E, Pogge D, Ahluvalia T, et al. Development and validation of a brief screening version of the Childhood Trauma Questionnaire. Child Abuse Negl. 2003;27(2):169-90.
3. Friis RH, Wittchen HU, Pfister H, Lieb R. Life events and changes in the course of depression in young adults. Eur Psychiatry. 2002;17(5):241-53.
4. Maier-Diewald W. Die Münchner Ereignisliste (MEL): Anwendungsmanual: Max-Plank-Institut für Psychiatrie; 1983.
5. Levine ME, Lu AT, Quach A, Chen BH, Assimes TL, Bandinelli S, et al. An epigenetic biomarker of aging for lifespan and healthspan. Aging (Albany NY). 2018;10(4):573-91.
6. Lu AT, Quach A, Wilson JG, Reiner AP, Aviv A, Raj K, et al. DNA methylation GrimAge strongly predicts lifespan and healthspan. Aging (Albany NY). 2019;11(2):303-27.
7. Belsky DW, Caspi A, Houts R, Cohen HJ, Corcoran DL, Danese A, et al. Quantification of biological aging in young adults. Proc Natl Acad Sci U S A. 2015;112(30):E4104-10.
8. Belsky DW, Caspi A, Arseneault L, Baccarelli A, Corcoran DL, Gao X, et al. Quantification of the pace of biological aging in humans through a blood test, the DunedinPoAm DNA methylation algorithm. Elife. 2020;9.
9. Horvath S. DNA methylation age of human tissues and cell types. Genome Biol. 2013;14(10):R115.
10. Wittchen H-U PH. DIA-X-Interviews: Manual für Screening-Verfahren und Interview; Interviewheft. 1997.
11. Houseman EA, Accomando WP, Koestler DC, Christensen BC, Marsit CJ, Nelson HH, et al. DNA methylation arrays as surrogate measures of cell mixture distribution. BMC Bioinformatics. 2012;13:86.
12. Houseman EA, Molitor J, Marsit CJ. Reference-free cell mixture adjustments in analysis of DNA methylation data. Bioinformatics. 2014;30(10):1431-9.
13. Conway JR, Lex A, Gehlenborg N. UpSetR: an R package for the visualization of intersecting sets and their properties. Bioinformatics. 2017;33(18):2938-40.
